# Charities of Physicians,—Hospitals,—Free Dispensaries, Etc.,—Their Influence upon the Profession and Public

**Published:** 1877-11

**Authors:** 


### EDITORIAL.

CHARITIES OF PHYSICIANS—HOSPITALS—FREE DISPENSARIES—FREE EYE AND EAR INFIRMARIES—ADVICE-TO-THE-POOR-GRATIS, AND OTHER SIMILAR "INSTITUTIONS"—THEIR INFLUENCE UPON THE PROFESSION AND PUBLIC.

Care of the sick and disabled is one of the first instincts and evidences of humanity, and too much cannot be said in commendation of the christian charity which fills the civilized world with Asylums, Hospitals, Homes for the poor, Infirmaries and similar benevolent institutions. All hail! to a sympathetic, generous, liberal regard for the deserving poor and disabled; but our charities even may be abused, overtaxed, unworthily applied, falsely named, selfishly and dishonestly conducted. Benevolence and charity are words for public approbation, but may cover actions more truthfully designated by other names. Duty, benevolence, and charity, are terms which often pass with the general public for a great deal more than they really represent.

We are under every obligation to take care of the insane, the poor, the sick, the aged and the generally disabled, but in caring for the insane we are not under obligation to take care of a much greater number who are of sound mind and capable of self support. As citizens, as physicians, as sympathisers with human suffering and poverty we are to remember the poor; as taxpayers we have a right to inquire into the facts of the situation, and as physicians to inquire into the justice and validity of the claim. As taxpayers we are obliged to pay two dollars for the privilege of giving one dollar to the poor, while as physicians, we are expected to not only care for the sick poor but a much greater number who want to save the expense of medical attendance for investment in much more questionable luxuries. As physicians we are greatly imposed upon, and as taxpayers cheated and robbed. Our hospitals are built and conducted upon the expectation that every expense is to be met either from the profits of the business, or contributions from the benevolent, except *one*. *Medical attendance* alone is left for gratuitous supply, and so far as hospital patients proper are concerned this is not to be complained of, but county paupers have no claims upon physicians' gratuitous care in hospitals or any where else. The Poor Department furnish them with physicians who are paid for their services. These are of course young men, who are not greatly experienced, and who are not wholly employed in care of better patrons, but difficult and obscure cases of disease or dangerous injury are sent to the hospitals, with the expectation of better professional care as well as general attention; every necessity being provided for by a general tax, except the medical attendance. There may be considerations of return, and so far perhaps there is but little cause of complaint, but a step farther. United States Marines deposit their money in custody of Government. Hospitals bid for their care, they compute expense upon the gratuitous care of Surgeons, and thus in Buffalo take the job at simple cost of food and medicine; in Buffalo at $4.99; Wheeling, Va., $7.00 per week. In Wheeling for instance there is less Hospital competition, and the Surgeon takes the government job, re-letting the general care to Hospital at $4.00, and making $3.00 per week for attendance. The hospitals should be obliged to reckon Surgeons' fees as an item of expense, and thus while receiving the same as now themselves, be able to pay Surgeons their fees; it is a manifest abuse of professional liberality to do otherwise, but this is no fault of hospitals. Physicians are alone responsible for the omission, medical service is so abundant as to have no market value. But lest we occupy too much space in representing conditions of which physicians have a right to complain, and are under obligations to correct, and really ought in justice to themselves and the profession to correct, we will pass to the "Free Infirmary Dispensary and advice-to-the-poor-gratis" Institutions of which we boast so largely. We do not propose to tell their number or names, do not care to give them a free advertisement, except to say of them as is said in other cities "they are an intolerable nuisance." Some uninformed will inquire "now why?" In Buffalo the expense of caring for the poor is supposed to justly rest upon all--to belong to a general tax. With that view nine Physicians and two Homeopaths are employed and paid to attend all the poor people who call upon them. They are distributed in all sections, and therefore within easy reach. To prevent imposition orders are to be furnished from Poormaster, so that "gratis to the poor" in our dispensaries means nothing more than an anonymous advertisement of some doctors who have little to do but advertise, and hope with this thin web to catch now and then a patient, whom they think has too good clothes for a poor-house, by representing to them that they have serious *diseases*, requiring more attention than can be given in dispensary infirmary hours, and which for small fees they will carefully investigate and treat at their private offices, No.—— ——street. This too might be all well enough, since so many share in the stock, but Supervisors are induced under representation of charity and great work to offer the prize of what medicine is required as inducement to those attracted by the sign of "*free*" to acccept advise gratis. It would seem that such advice from "experts"(?) in the various departments of Medicine and Surgery might be accepted by the poor without being paid for it from the general tax, but all these free institutions draw upon the poor department for support, or intend to do so.

Physicians are liberal to a fault, they all give advice cheerfully and freely to those who ask and seem to require it. Free to the poor might as truthfully be placed upon every physicians sign, as upon the door of dispensaries, infirmaries, &c., &c. It is a reflection upon the universal liberality of physicians to ask the poor department to hire poor people to patronize "the-free-to the-poor institutions."

In conclusion we have a word to say of the influence of all this, upon the profession and public. The respectability of physicians is lessened, the value of their ministrations diminished and the rank they should hold in society is lowered. The public are made to feel that physicians are very anxious to have them take their advice, possibly even their medicine, while the true standard of an honest high minded physician should be such as to impress the families of even the affluent that it is a compliment to them and a favor to the family rather than the physician. One of our patients often says proudly "I was attended in 1870, by Mr. Wilson of London," which is an illustration of the spirit we would have physicians cultivate.

Though we base our remarks mainly upon the situation in Erie county, the same state of affairs prevails elsewhere, two thirds of the people of London are said in a recent English Journal, to receive gratuitous medical attendance, the great majority of which are abundantly able to render adequate compensation.

